# Skin Diseases and Syphilis

**Published:** 1895-12

**Authors:** 


### SKIN DISEASES AND SYPHILIS.

EDITED BY DR. M. B. HUTCHINS.

### NOTES ON CHANCRES.

Hutchinson, Jonathan. (*Archives of Surgery,* January, 1895.)

Mr. Hutchinson speaks in this article of chancres recurring in the site of old sores without fresh venereal inoculation. He first treats of the theory of herpetic chancres. Herpes on the prepuce and glans recurring frequently is much more common in patients who have had syphilis than in others. The herpes may follow the non-indurated as well as the hard sore; is usually of short duration, disappearing irrespective of treatment in the course of a week or ten days; it is not benefited by mercury, but may be prevented by the use of arsenic.

Some cases do not get well so quickly, and occasionally degenerate into chronic ulcers, very difficult to distinguish from the results of recent infection. The herpes is due to local nervous disturbance in tissues which have been affected by the syphilitic taint. It may follow venereal excitement. In a case mentioned by the author, new sores developed on the penis three years after an attack of syphilis, and apparently without any possibility of recent infection. Some showed definite induration, and the patient was under treatment for two months. Mercury did not act well.

On the after-marriage chancre. These occur in cases where there is no evidence of remaining syphilitic taint than the local sore on the genital organ. Sometimes exactly on the site of former chancres, sometimes not so. They never cause enlarged glands, nor are they followed by secondary symptoms, but occasionally prove infective to the wife, and Mr. Hutchinson believes that in all cases in which young wives acquire syphilis from apparently healthy husbands, sores of this kind have intervened.

The author thinks that the liberal sexual intercourse often following marriage, usually preceded by a long interval of abstinence, is prone to relight old chancres. He has seen so many under these circumstances that "it is impossible to believe that they are matters merely of coincidence, or that in all the patient is guilty of concealment." As a rule, a man is not likely to expose himself to the risk of contracting fresh disease just on the eve of his marriage.—*Med. Chronicle.*

---

### PSEUDO CHANCRE.

Reinfectio syphilitica does occur, though the recorded cases that are entirely trustworthy are very few indeed. Analysis shows that most of the alleged cases are open to grave doubt, and some of them are manifestly errors of diagnosis.

The following lesions may simulate chancre.

*a.* Artificial indurations caused by irritants applied to simple lesions.

*b.* Nodular lymphangites, as occur in gonorrhea.

*c.* Scabies, where penile lesions are the rule.

*d.* Secondary indurations at the site of the initial lesion (Fournier's pseudo-chancre).

*e.* Secondary syphilitic papules or tubercles situated upon the genitals.

*f.* Ulcerative gummata of the genitals.

*g.* Epitheliomata of the genitals.

Two such cases have recently come under the author's observation. In the first one a non-specific sore was irritated with cauterisants until it exactly resembled a sclerosis with central ulceration, and was so diagnosticated by competent authorities. Nevertheless, it healed up under local treatment alone ; and until now, two years after date, no secondary symptoms have appeared.

The other case was one of gumma of the penis in a subject in the tertiary stage of syphilis. The lesion resembled an initial one very closely, and was at first regarded as such ; but a close examination showed the presence of evidence of past specific disease, and this was confirmed by the history. The entire lesion melted away under the iodide of potash.

Conclusions:

1. There is no characteristic sign, and no characteristic combination of signs, that enables us to diagnosticate a chancre from the lesion alone.

2. Only the advent of other syphilitic symptoms enables us to form an opinion as to the presence of systemic infection.

3. Almost all the alleged cases of syphilitic reinfection are of doubtful validity; and most of them are pseudo-chancres belonging to one or other of the above varieties.—WM. S. GOTTHEIL, M.D., *N. Y. Med. Journal.*

